# Biological Detoxification of Aflatoxin B_1_ by *Enterococcus faecium* HB2-2

**DOI:** 10.3390/foods13121887

**Published:** 2024-06-15

**Authors:** Jiangtao Feng, Ling Cao, Xiaoyan Du, Yvying Zhang, Yanxia Cong, Junbo He, Weinong Zhang

**Affiliations:** 1 Key Laboratory for Deep Processing of Major Grain and Oil, Ministry of Education, College of Food Science & Engineering, Wuhan Polytechnic University, Wuhan 430023, China; jtfeng@whpu.edu.cn (J.F.); junb112he@whpu.edu.cn (J.H.); 2 Hubei Key Laboratory for Processing and Transformation of Agricultural Products, Wuhan Polytechnic University, Wuhan 430023, China; 3 Engineering Research Center of Lipid-based Fine Chemicals of Hubei Province, Wuhan 430023, China

**Keywords:** aflatoxin B_1_, biological degradation, *Enterococcus faecium*, peanut meal

## Abstract

Aflatoxin B_1_ (AFB_1_) contamination in food and feed is a global health and economic threat, necessitating the immediate development of effective strategies to mitigate its negative effects. This study focuses on the isolation and characterization of *Enterococcus faecium* HB2-2 (*E. faecium* HB2-2) as a potent AFB_1_-degrading microorganism, using morphological observation, biochemical profiling, and 16S rRNA sequence analysis. An incubation of *E. faecium* HB2-2 at 32 °C for 96 h in a pH 10 nutrient broth (NB) medium resulted in a remarkable degradation rate of 90.0% for AFB_1_. Furthermore, *E. faecium* HB2-2 demonstrated 82.9% AFB_1_ degradation rate in the peanut meal, reducing AFB_1_ levels from 105.1 to 17.9 μg/kg. The AFB_1_ degradation ability of *E. faecium* HB2-2 was found to be dependent on the fermentation supernatant. The products of AFB_1_ degradation by *E. faecium* HB2-2 were analyzed by liquid chromatography–mass spectrometry (LC-MS), and a possible degradation mechanism was proposed based on the identified degradation products. Additionally, cytotoxicity assays revealed a significant reduction in the toxicity of the degradation products compared to the parent AFB_1_. These findings highlight the potential of *E. faecium* HB2-2 as a safe and effective method for mitigating AFB_1_ contamination in food and feed.

## 1. Introduction

Mycotoxins are secondary metabolites synthesized by many fungi that commonly infest plants, food, and feed, including *Aspergillus*, *Penicillium*, and *Fusarium*. Mycotoxins typically contaminate agricultural commodities throughout harvest and storage periods, including peanuts, corn, cottonseed, and tree nuts [1]. More than 400 mycotoxins have been detected worldwide, according to reports [2]. Mycotoxins most frequently encountered are ochratoxin, aflatoxins, zearalenone, deoxynivalenol, and fumonisin [2,3]. Their adverse effects are manifold and include reproductive toxicity, immunological toxicity, genetic toxicity, hepatorenal toxicity, and cardiac toxicity [4,5,6]. Aflatoxins (AFs) are highly toxic mycotoxins synthesized primarily by filamentous fungi such as *Aspergillus flavus*, *Aspergillus nomius*, and *Aspergillus parasiticus* [7,8]. Aflatoxins significantly contaminate many types of grains and are widely present in both human meals and animal feed [8]. AFs are known for their severe and long-lasting harmful effects, with different symptoms including nausea, vomiting, and stomach discomfort, as well as their ability to cause cancer, birth defects, and liver damage [9]. Several distinct AFs architectures have been documented, including aflatoxin B_1_ (AFB_1_), aflatoxin B_2_, aflatoxin G_1_, and aflatoxin G_2_. AFB_1_ stands out as the most notable and dangerous variety due to its ability to cause birth defects, genetic mutations, and cancer. As a result, the International Agency for Research on Cancer (IARC) classified it as a Group I carcinogen in humans [10].

The potential contamination of agricultural commodities and crops by AFB_1_ is a significant issue, especially regarding peanuts and their products. Peanut meal, a by-product derived from the oil extraction process, is extensively utilized as animal feed for livestock, poultry, and aquaculture [11,12]. However, the presence of AFB_1_ in peanut meal poses a significant risk to animal health and can potentially accumulate in the human body through the food chain. Thus, the development of effective and environmentally friendly techniques to eliminate AFB_1_ in peanut meal is of utmost importance.

The degradation and removal of AFB_1_ from food and feed materials have been extensively studied using various methods, including physical techniques such as heat, absorption, irradiation, and ultraviolet light [9,13,14], chemical measures such as ozonization, ammoniation, and sodium hydroxide [15,16,17], and biological approaches involving microorganisms and enzymes [18,19,20]. In recent years, biological approaches such as microbial degradation and enzymatic degradation have gained significant interest due to their high efficiency, cost-effectiveness, and animal-friendly nature. Bacteria such as *Rhodococcus erythropolis* [21], *Myxococcus fulvus* [22], and *Pseudomonas* spp. [23] have been reported to be effective in removing AFB_1_ from food and feed. However, the majority of these documented strains have not been employed for the detoxification of aflatoxin in food or feed materials due to concerns over biosafety during actual production. *Enterococcus faecium* (*E. faecium)* is a bacterium commonly found in the gastrointestinal system of animals. *E. faecium* is reported as a probiotic and crucial feed supplement that is extensively utilized in livestock production [24,25]. While it has been reported that *Enterococcus faecium* strains showed 19.3 to 37.5% degradation ability of AFB_1_ [26], the research did not investigate the degradation of AFB_1_ specifically in the actual sample like peanut meal, its byproducts, and the potential toxicity associated with it. Therefore, it is imperative to thoroughly examine the degrading activity and the mechanism of AFB_1_ in peanut meal.

The main objectives of this study were the following: (1) to screen and isolate potential strains exhibiting efficient detoxification of AFB_1_ and then identify the optimum strain through physiological, morphological, and 16s rRNA gene sequencing analysis; (2) to enhance the detoxifying potential of this promising strain by refining the fermentation conditions; and (3) to investigate the potential mechanism of AFB_1_ degradation by the strain, as well as the toxicity of the degradation products.

## 2. Materials and Methods

### 2.1. Chemicals and Culture Media 

The AFB_1_ standard, with a purity of 98%, was acquired from Bailing Wei Technology Co., Beijing, China. It was dissolved in methanol at a concentration of 10 mg/kg and utilized as the standard stock solution for this work. The solution was maintained at −20 °C, protected from light. HepG2 and RAW264.7 cells, and DMEM medium were purchased from Beijing Solarbio Science & Technology Co.,Ltd. (Beijing, China). 3-(4, 5-Dimethylthiazol- 2-yl)-2,5-diphenyltetrazolium bromide (MTT) was procured from Invitrogen (Waltham, MA, USA). The coumarin used in this study was of analytical purity and was sourced from Shanpu Chemical Co., Ltd., located in Shanghai, China. Methanol (with high chromatographic purity), acetonitrile (with high chromatographic purity), and unless stated, all the other reagents are purchased from procured from Sinopharm Chemical Reagent Co., Ltd., located in Shanghai, China, like dipotassium hydrogen phosphate, potassium dihydrogen phosphate, Protease K, hydrochloric acid, sodium hydroxide, sodium chloride, and so on. The nutrient broth (NB) medium is prepared by dissolving 1 g of yeast powder (Biosharp life science, Hefei, Anhui, China), 5 g of peptone (Biosharp life science, Hefei, Anhui, China), 15 g of sucrose, and 3 g of beef paste in 1000 mL of ultrapure water. The mixture is then autoclaved at a temperature of 121 °C for 20 min. The Coumarin medium is a minimal salt media and prepared according to Wang et al. [27]. It consists of the following ingredients: (NH_4_)_2_SO_4_ (5.0 g), KHPO_4_ (2.5 g), MgSO_4_ (1.0 g), NaHPO_4_-12H_2_O (0.5 g), CaCl_2_ (0.1 g), agar (20 g), and coumarin (1 g). All these components are dissolved in 1000 mL of water and sterilized at a temperature of 121 °C for 20 min.

### 2.2. Isolation of AFB_1_-Degrading Strains

A total of 50 soil samples were gathered from grassland soil (Wuhan, China, 2022), rubbish (Wuhan, China, 2022), mildewed peanut (Wuhan, China, 2022), and peanut fields (Huanggang, China, 2022). The samples were collected in sterile plastic sampling bags and stored in an icebox to preserve the original microbial activity. They were then transported directly to the laboratory for the purpose of screening aflatoxin-degrading bacteria. The experimental approach was conducted following the stated methodology with minor alterations [27]. 1 g soil sample was mixed well with 10 mL of sterile water. The mixture was then placed in a rotary shaker and incubated at a temperature of 28 °C for 3 h. Serial dilutions of the bacterial solution were performed, starting from a 10^−1^ dilution and going up to a 10^−8^ dilution, following the specified method with any necessary adjustments. Subsequently, 100 µL dilution was evenly distributed over the solid plates containing coumarin medium. The plates were then incubated for 12–24 h at a temperature of 28 °C. The color and shape of colonies on plates were examined using a microscope, and then strains were selected and isolated by different colors and shapes. Subsequently, to obtain strains which could grow on the NB agar plate using coumarin as the only carbon source, each of the isolated strains was incubated into a NB agar plate containing 0.1% coumarin at a temperature of 28 °C for 24 h. Then, the isolated strains which could grow on the NB agar plate containing coumarin were streaked successively on a plate to obtain pure culture, which were subsequently preserved in 25% glycerol at a temperature of −80 °C.

### 2.3. Aflatoxin Degradation Assay

The degradation of AFB_1_ was conducted in liquid cultures following the procedures outlined, with minor adjustments [28]. A single colony of the isolated strain was chosen and then inoculated into a liquid NB media and incubated at 28 °C and placed on a rotary shake (200 rpm) for 12 h. Subsequently, 1 mL of the fermentation liquid of strain HB2-2 was mixed with 9 mL of fresh NB liquid medium at a 10% inoculation rate. Subsequently, 120 µL of the standard stock solution of AFB_1_ (10 mg/kg) was mixed with the fermentation broth of strain HB2-2, and AFB_1_ mixed with NB medium without HB2-2 strain was taken as the control group. The resulting combination was then subjected to incubation in a shaking incubator at 200 rpm, maintaining a temperature of 28 °C for 48 h. Following a 2-day degradation process, the remaining AFB_1_ was extracted from the fermentation mixture or NB medium (control group), and subsequently quantified using high performance liquid chromatography (HPLC) as described in Section 2.4. The concentration of AFB_1_ in the control group (NB medium without HB2-2 strain) was quantified 113.0 μg/kg, the corresponding recovery rate was 94.17%.

### 2.4. Extraction and Detection of Residual AFB_1_

The experimental method of AFB_1_ extraction and detection was conducted according to the determination of aflatoxins B and G in food of national food safety standard (GB 5009.22—2016) [29]. The standard specifies that the limit of detection (LOD) of AFB1 for this method is 0.03 μg/kg, and the limit of quantification (LOQ) is 0.1 μg/kg. The remaining AFB_1_ was extracted from the fermentation broth using immunoaffinity columns manufactured by zhongke huiren Science and technology Co., Ltd. (Beijing, China) for quantitative analysis. The product specification of immunoaffinity columns indicates that the recovery rate of AFB_1_ is greater than 90%, while in this study, the recovery rate of AFB_1_ is 94.17%. The process involved the removal of microbial cells from the fermentation mixture through centrifugation at 5000 rpm for 10 min, obtaining the supernatant. Subsequently, 2 mL of the fermentation supernatant was mixed with 8 mL of aqueous methanol (*V*/*V*, Methanol/water = 7:3) and placed in a shaker (200 rpm) at room temperature for 20 min. The mixture was then centrifuged at 5000 rpm for 10 min, leading to the separation of the precipitation, which was discarded, and the retention of the supernatant solution. A total of 4 mL of the supernatant solution was combined with 23 mL of PBS/Tween-20 (1%) buffer. Subsequently, the mixture was gradually flowed through the immunoaffinity column. The residual AFB_1_ was then eluted using 1 mL of methanol, and the resulting solution was filtered through a 0.22 µm organic ultrafiltration membrane. The quantity of AFB_1_ was measured using an HPLC system (Agilent 1260 II, Palo Alto, CA, USA) that had an AQ C_18_ reverse phase column (length 150 mm, internal diameter 4.6 mm, and pore size 5 μm). A fluorescence detector (Agilent, Palo Alto, USA) was used, along with a post column derivatization system (Photochemical Derivative Instrument type PHRED-HR, zhongke huiren Science and technology Co., Ltd. Beijing, China) to enhance the sensitivity of fluorescence. The injection volume was 10 μL, the column temperature was kept at 40 °C. The mobile phase consisted of a mixture of water, methanol, and acetonitrile in a ratio of 55:35:10 (*v*/*v*/*v*) and flowed at a rate of 0.9 mL/min. The spectra’s excitation and emission wavelengths were 360 and 440 nm, respectively [29,30]. The experiments were replicated three times, the HPLC spectrogram of AFB_1_ in the supporting information Figure S1 (retention time was 18.05 min), and the standard curve of AFB_1_ was showed in Figure S2 of the support information. The degradation activity of AFB_1_ was determined using the following formula.

Degradation rate of AFB_1_ (%) = (1 − A_1_/A_2_) × 100%

where A_1_ represents the amount of residual AFB_1_ in the fermentation supernatant solution treated with HB2-2 strain, and A_2_ is the control and represents the amount of AFB_1_ in the NB media without HB2-2 strain.

### 2.5. Identification of the HB2-2 Strain

In order to determine the morphological and biochemical traits of the isolated HB2-2 strain, a series of physiological and biochemical tests were conducted following the reported procedure [15]. The strain HB2-2, which was kept separate from other strains, was inoculated into NB solid medium using a slanted line technique. The culture was then incubated at a temperature of 28 °C for 24 h. A small amount of bacteria were selected for Gram staining using a Gram Staining Kit (Biosharp life science, Hefei, Anhui, China), and the cellular structure and staining outcomes were examined using a microscope. In addition, physiological and biochemical tests were carried out followed by Schaad [31]. Scanning electron microscopy was used to better observed the cellular morphology of the bacteria. The bacterial strain HB2-2 was grown in an incubator shaker at a speed of 200 rpm and a temperature of 28 °C for 24 h. The strain HB2-2 cells were collected by centrifugation at a speed of 10,000 rpm for 10 min and washed with PBS three times. The shape of the HB2-2 cells was fixed by adding 2.5% glutaraldehyde at a temperature of 4 °C overnight, followed by three washes with PBS as described in previous studies [32,33]. The HB2-2 cells were dehydrated using a succession of gradient alcohols (30%, 50%, 70%, 90%, 100%) and then freeze-dried, following the method described in previous publication [34,35]. A small amount of strain HB2-2 sample was dispersed onto the conductive adhesive surface. Subsequently, the sample which had not adhered to the adhesive was removed using an ear wash ball. Then, the treated surface was given a spray gold coverage for 30 s. The cellular morphology of strain HB2-2 were subsequently observed using a Field Emission Scanning Electron Microscope S-4800 (Hitachi, Tokyo, Japan).

In addition, the analysis of the 16S rRNA gene sequence was conducted using the methods described in the literature [18,36]. In summary, the whole DNA from strain HB2-2 was obtained using a Bacterial DNA extraction kit (SBS Gene, Shanghai, China), following the manufacturer instruction, and then amplified using Taq PCR Master Mix kit (SBS Gene, Shanghai, China) with universal primers specific to bacterial 16S rDNA, 7F 5′-CAGAGTTTGATCCTGGCT-3′, and 1540R 5′-CAGAGTTTGATCCTGGCT-3′ [37]. The PCR protocol was established with the following conditions: an initial denaturation step at 94 °C for 4 min one cycle, followed by denaturation at 94 °C for 45 s, annealing at 55 °C for 45 s, extension at 72 °C for 1 min, and a total of 30 cycles. A final extension step was performed at 72 °C for 10 min. The amplified products underwent sequencing and the sequences were compared by Blast using GenBank database in NCBI. MEGA6.0 software was applied to construct phylogenetic tree for sequences with high homology [15,38].

### 2.6. Impact of Various Fermentation Conditions on the Degradation of AFB_1_

The AFB_1_-degrading activity of HB2-2 strain was influenced by various fermentation conditions, such as amounts of the strain, fermentation time, temperature, and pH. Therefore, various AFB_1_ degradation studies were conducted under different culture conditions to determine the optimal parameters for AFB_1_ degradation of strain HB2-2. The strain HB2-2 was incubated in 20 mL NB medium at 200 rpm at 28 °C for 12 h reaching a cell density to OD600 = 1.2. Subsequently, 1 mL of the fermentation liquid of strain HB2-2 was added to 9 mL of fresh NB medium, and mixed with 120 µL of the standard stock solution of AFB_1_ (10 mg/kg). The mixture was then incubated in different culture conditions. The control group was taken as the AFB_1_ mixed with NB medium without HB2-2 strain, and the concentration of AFB_1_ in the control group was quantified 113.0 μg/kg. To determine the ideal fermentation temperature, the mixture was incubated at different temperatures (20, 24, 28, 32, and 35 °C) for 48 h. After incubation, the fermentation mixture was collected and the remaining AFB_1_ was extracted and analyzed using HPLC. The impact of varying initial inoculum amounts of strain on the breakdown of AFB_1_ was assessed by adding varied amounts of fermentation liquid (1%, 5%, 10%, and 20%) to fresh NB medium. The medium was then spiked with AFB_1_ and cultured at a temperature of 32 °C for 48 h. The optimal pH was obtained by altering the pH (1 mole/L HCL or 10 mole/L NaOH) of the NB media to a range of 2.0–11.0 using a pH meter (METTLER TOLEDO, Zurich, Switzerland) before autoclaving, and incubating it at a temperature of 32 °C for 48 h. To investigate the impact of fermentation period on the breakdown of AFB_1_, the fermentation mixture of strain HB2-2 was incubated with AFB_1_ at a temperature of 32 °C for various durations of 24, 48, 72, 96, and 120 h, The control group was taken as the AFB_1_ (118.3 μg/kg) mixed with NB medium without HB2-2 strain.

### 2.7. Degradation of AFB_1_ by Strain HB2-2 in Peanut Meal

The strain HB2-2 was further applied to eliminate AFB_1_ in moldy peanut meal. The moldy peanut meal was collected and dried in oven at 65-70 °C, and ground with a blender. The fermentation liquid of strain HB2-2 was prepared as the mentioned procedures in Section 2.3, and the peanut meal contaminated AFB_1_ (105.1 µg/kg) was mixed with fermentation liquid of strain HB2-2 at different material-to-liquid ratio (peanut meal/fermentation liquid, 1:1, 1:5, 1:10, 1:15, 1:20 and 1:30). The mixtures were adjusted pH to 10 and incubated at 32 °C for 96 h, under the post-optimized fermentation conditions obtained in Section 2.6, and the residual AFB_1_ was extracted and analyzed by HPLC.

### 2.8. The Different Cellular Fractions of Strain HB2-2 on AFB_1_ Degradation

In order to assess the impact of various cellular components of the isolated strain HB2-2 on the breakdown of AFB_1_, culture supernatants, cell suspensions, and cell extracts were generated following the methodology outlined in a previous work [39]. The isolated strain HB2-2 was cultured at a temperature of 28 °C for 48 h in NB medium. The culture supernatant and cell pellet were collected and isolated using centrifugation at a speed of 10,000 rpm for 20 min at a temperature of 4 °C. Following centrifugation, the culture supernatant was filtered using a sterile 0.22 μm pore size filter and utilized for subsequent studies. The bacterial pellet was rinsed twice and resuspended in phosphate buffer, serving as the cell resuspension solution for further studies. To acquire the intracellular extracts, a part of the resuspended cell was disrupted using an Ultrasonic Cell Disruption System (Scientz, Ningbo, China) in an ice bath. The disrupted cell was then centrifuged at a speed of 10,000 rpm for 20 min at a temperature of 4 °C. Finally, the extract was filtered through a sterile filter with a pore size of 0.22 μm [40]. The culture supernatants, cell suspensions, and intracellular extracts were mixed with AFB_1_, respectively. AFB_1_ mixed with NB medium without HB2-2 strain was taken as the control group, The groups were subjected to incubation at a temperature of 32 °C for 96 h in order to assess the degradation capacity of the various fractions. The remaining AFB_1_ was then extracted and quantified using HPLC, as previously described. And the concentration of AFB_1_ in the control group was quantified 113.0 μg/kg.

### 2.9. The Impact of Heat Inactivation, Proteinase K, and Proteinase K Combined with Sodium Dodecyl Sulfate (SDS) Treatments on the AFB_1_ Degradation Activity of the Fermentation Supernatant

In order to ascertain if the degradation of AFB_1_ was facilitated by proteins or other metabolites in the fermentation supernatant, additional experiments were conducted on the culture supernatant treated with heat, proteinase K, and SDS + proteinase K, following established protocols [41,42,43]. Proteins undergo denaturation and deactivation when exposed to elevated temperatures [27]. Protease K has the ability to hydrolyze and deactivate proteins, while SDS can deactivate proteins by disrupting their tertiary and quaternary structures [44,45]. For the process of heat treatment, the liquid remaining after fermentation was subjected to boiling (about 98 °C) for 1 h using a water bath. During the proteinase K treatment, a concentration of 1 mg/mL of proteinase K was mixed with the bacterial fermentation supernatant and incubated at a temperature of 55 °C for 3 h. To perform proteinase K and SDS treatment, a mixture of 1 mg/mL proteinase K and 1% SDS was subjected to simultaneous treatment at a temperature of 55 °C for 3 h. Each fermentation supernatant was enriched with 200 ng/mL of AFB_1_ and then incubated at a temperature of 32 °C for 96 h. The control was made using the fermentation supernatant that was not treated. The retrieval and examination of AFB_1_ were carried out according to the procedures outlined in Section 2.4.

### 2.10. Analysis and Identification of Degraded Products

A total of 1 mL of fermentation liquid of strain HB2-2 was combined with 9 mL of NB medium. Subsequently, AFB_1_ storage solution was added until the final concentration reached 0.2 µg/mL. The mixture was subsequently incubated at a temperature of 32 °C for 48 h. The fermentation supernatant underwent centrifugation at a speed of 10,000 rpm for 10 min at a temperature of 4 °C. It was then filtered using a sterile filter with a pore size of 0.22 μm from Tianjin Jinteng experimental Equipment Co., LTD (Tianjing, China). The degradative compounds of AFB_1_ by HB2-2 strain were complex mixture, for LC-MS study. Then, a preliminary purification was performed using the immunoaffinity columns, which were used in the AFB_1_ extraction experiment. Subsequently, the complex mixture of AFB_1_ degradative compounds was passed through an immunoaffinity column, following the procedure outlined in Section 2.4. The degraded products were gradually separated by the addition of methanol, and the resulting solution was evaporated under N_2_ gas at a temperature of 50 °C. The desiccated substance was diluted in 1 mL of methanol, then filtered using a 0.22 μm microporous filter membrane. The resulting concentrated solution of degradation products was utilized for subsequent analysis. The control group was treated identically to the experimental group, with no exposure to strain HB2-2. The investigation of AFB_1_ and its degradation products was conducted utilizing the Ultimate 3000 UHPLC—Q Exactive LC-MS System, manufactured by Thermo Scientific in the United States. In the process of chromatographic analysis, a volume of 5 μL of the test sample was inoculated onto an Atlantis T3 C_18_ column with dimensions of 100 mm × 3.0 mm and a particle size of 3.0 μm, manufactured by Waters Corporation in the United States. The mobile phase consisted of a 0.1% formic acid water solution and acetonitrile in a 1:1 ratio, and was performed at a consistent flow rate of 0.4 mL/min. The column temperature was maintained at 30 °C. The Thermo Scientific Q Exactive mass spectrometer system was utilized for the purpose of conducting mass spectroscopy. The specific parameters used were electro-spray ionization, ion source HESI, positive polarity (ES^+^), capillary voltage of 3.8 kV, and capillary temperature of 320 °C. The mass spectrometer was operated using the Fullms/dd-ms2 scan mode. An electrospray ionization interface detector was utilized to measure the mass of the separated products within the mass range (m/z) of 50–600. The obtained specific m/z values were identified based on the literature representing the AFB_1_-degraded products by various microorganisms. Furthermore, the hypothetical degradation pathway of AFB_1_ was speculated based on the degraded products [44].

### 2.11. Cytotoxicity Assay of the Degradation Products of AFB_1_

The cytotoxicity of AFB_1_ and its degradation products were assessed by evaluating the cell viability of HepG2 and RAW264.7 cells [45,46]. The degradation products of AFB_1_ were isolated from the bacterial fermentation supernatant using an immunoaffinity column. The extracted compounds were then dried under N_2_ gas at 50 °C. The remaining degradation products were dissolved in 100 μL of DMSO and 10 mL of DMEM medium containing 10% FBS for subsequent studies. The cryopreserved HepG2 cells and Raw264.7 cells were revived, treated with trypsin for digestion, and cultured in DMEM medium (10% FBS) until they reached the logarithmic growth phase. Subsequently, they were transferred into 96-well plates at a density of 1 × 10^4^ cells per well and incubated at 37 °C under 5% CO_2_ for 24 h. The liquid in the 96-well plate was removed, and 100 µL of DMEM medium (10% FBS) containing degradation products of AFB_1_ was added. Subsequently, the 96-well plate was incubated at a temperature of 37 °C with a 5% CO_2_ for durations of 24 h, 48 h, and 72 h, respectively. The control group was incubated with 100 μL of DMEM medium per well. Afterwards, cell viability was assessed using the MTT method. Specifically, 10 µL of MTT solution (5 mg/mL) was added to each well and incubated for an additional 4 h. The supernatant was then withdrawn and 100 μL of DMSO was added, followed by gentle shaking for 10 min. The cell viability was assessed by quantifying the absorbance at 590 nm, and the rate of cell growth inhibition was computed using the following formula.

$$Cell\;Growth\;inhibition\;rate\%=\frac{A_{Control}-A_{Sample}}{A_{Control}}\times 100\%$$

The sample consisted of DMEM containing AFB_1_ or its degraded products, while the control consisted of DMEM without AFB_1_ or its degraded products.

### 2.12. Data Analysis

The tests were conducted in three replicates, unless otherwise specified. The data in this research were analyzed and graphed using Origin 7.5 software, and the findings were presented as the mean value plus or minus the standard deviation. One-way analysis of variance (ANOVA) and correlation analysis were employed to analyze the data. Duncan’s multiple range test was chosen to locate the differences between means, and a significance level of 5% (*p* < 0.05) was considered acceptable.

## 3. Results and Discussions

### 3.1. Screening of AFB_1_-Degrading Strains 

**Figure 1:**
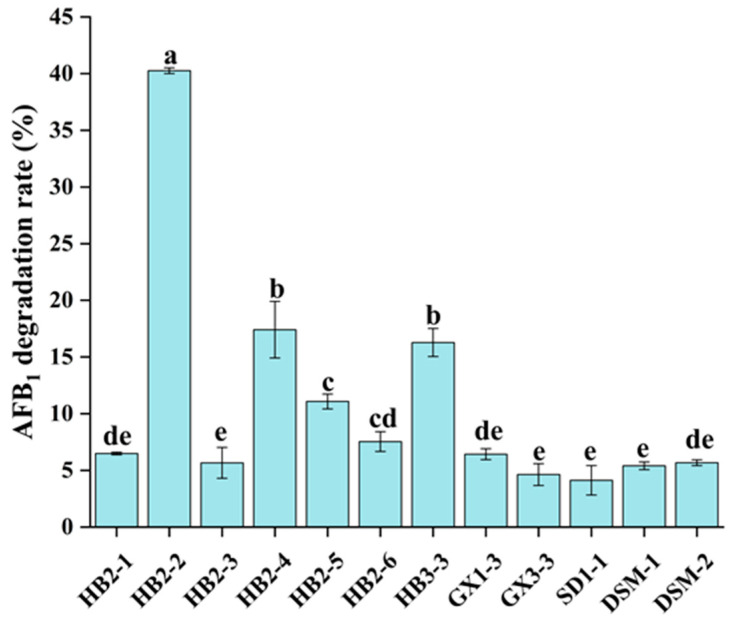
Degradation rate of AFB_1_ by isolated strains. Values in the bar graph represent the mean values ± SD of three independent experiments. Different lower-case letters in the same items indicate a significant result as determined by Duncan’s range test (*p* < 0.05).

In this investigation, a total of 50 soil samples were collected from grassland soil, garbage, mildewed peanut, and peanut fields. Coumarin serves as the fundamental molecular framework for all aflatoxins, and is regarded as a more cost-effective and safer alternative for a AFB_1_ degradation microbe. Consequently, coumarin media was employed to identify microorganisms capable of breaking down AFB_1_, using coumarin as the sole source of carbon according to a reported study [47]. The solution of soil samples was diluted and evenly distributed over a solid medium containing 0.1% coumarin. A total of 43 strains were isolated on the plate following incubation at 28 °C for 48 h. For AFB_1_-degrading strains screening, all the isolated 43 strains were incubated with AFB_1_ at a concentration of 113.0 μg/kg at 28 °C for 48 h, respectively. Additionally, the AFB_1_ degradation abilities of strains were presented in Figure 1, 12 strains showed AFB_1_ degradation ability to some extent, from 4.1% to 40.3%. Similarly, a total of 65 bacterial isolates were effectively screened from 119 biological samples utilizing 0.1% coumarin as the only carbon source [48]. In an early investigation, 43 single-colony bacterial isolates were isolated from 247 samples taken from different sources using coumarin media, and all of which were able to decrease AFB_1_ to variable degrees [49]. The bacterium HB2-2 isolated from soil sample demonstrated 40.3% AFB_1_-degrading activity and was selected for further research.

### 3.2. Physiological, Morphological and 16s rRNA Characterization of Strain HB2-2

**Figure 2:**
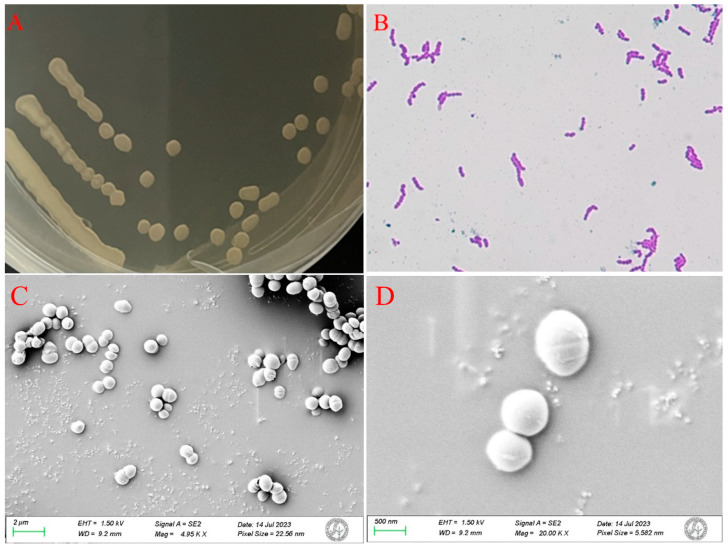
Identification of strain HB2-2 from morphologies. (**A**) Colony characteristics of the strain; (**B**) the cell morphology of Gram staining; (**C**,**D**) the bacterial fine structure observed by SEM.

The morphological, biochemical and 16s rRNA gene sequencing characteristics of the isolated strain HB2-2 were further examined. As shown in Figure 2A, the colonies of strain HB2-2 cultured on NB media exhibited a spherical morphology, characterized by well-defined borders and an opaque, creamy white appearance with a slightly lighter hue on the anterior side. They were elevated at the center, displaying a smooth surface and a wet texture, facilitating easy pick up, and the Gram stain of strain HB2-2 was positive, as shown in Figure 2B.

**Figure 3:**
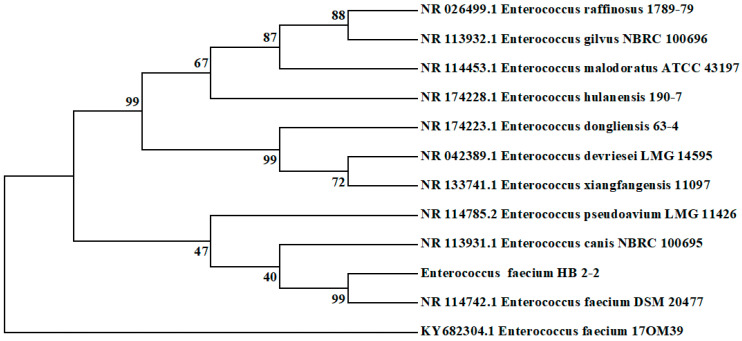
The phylogenetic tree constructed based on 16S rRNA gene.

Scanning electron microscopy (SEM) was utilized to study the fine structure of the strain HB2-2 (Figure 2C,D). The cells of strain HB2-2 were complete spheroids with a smooth surface. Furthermore, certain physiological and biochemical tests were carried out in Table 1. Subsequently, the online BLAST search of the 16S rRNA gene sequence of the bacteria HB2-2 demonstrated a 99% resemblance to *Enterococcus* faecium DSM 20477. A neighbor-joining phylogenetic tree was generated based on 16S rRNA gene sequencing (Figure 3). Finally, on the basis of morphological, physiological and investigation of the 16s rRNA gene sequence, the isolated strain HB2-2 was identified as *Enterococcus Faecium* (*E. faecium* HB2-2). The results were in keeping with a recent study, in which, two probiotic *E. faecium* M74 and *E. faecium* EF031 were reported to degrade AFB_1_ [26]. *Enterococcus faecium* is a member of lactic acid bacteria mostly found in nature, especially in foods and has various applications in the processing of some fermented dairy products, and which was reported as a probiotic used in food and feed to effectively improve animal immunity [1,16].

### 3.3. Effect of Fermentation Conditions on the Degradation of AFB_1_

**Figure 4:**
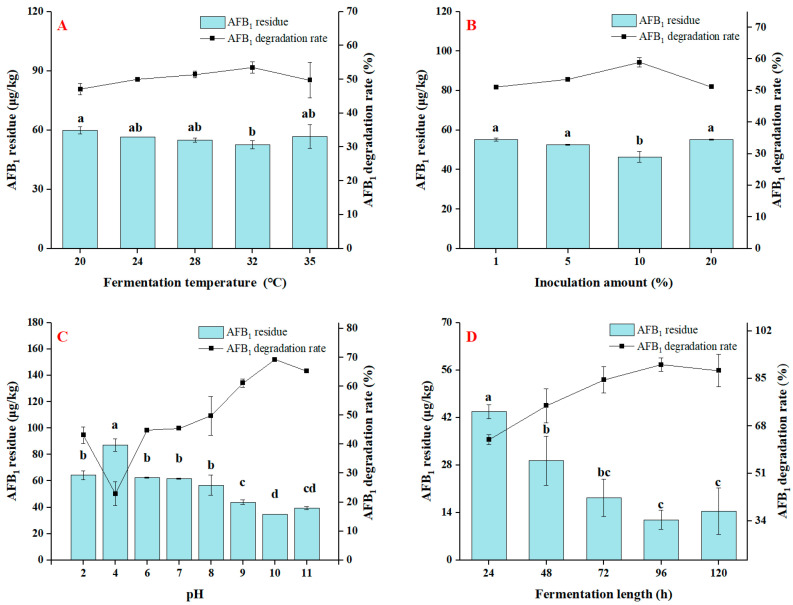
Effects of different fermentation conditions on the degradation of AFB_1_. (**A**) The AFB_1_ degradation activity of *E. faecium* HB2-2 incubated at different temperature (20, 24, 28, 32 and 35 °C); (**B**) the AFB_1_ degradation activity of *E. faecium* HB2-2 at varying initial inoculum amounts of strain (1%, 5%, 10%, and 20%); (**C**) the AFB_1_ degradation activity of *E. faecium* HB2-2 incubated at various pH values of NB medium (pH 2, 4, 6, 7, 8, 9, 10 and 11); (**D**) the AFB_1_ degradation activity of *E. faecium* HB2-2 incubated for different times (24, 48, 72, 96 and 120 h). Values in the bar graph represent the mean values ± SD of three independent experiments. Different lower-case letters in the same items indicate a significant result as determined by Duncan’s range test (*p* < 0.05).

To optimize the degradation efficiency of the strain, varied parameters of temperature, initial inoculum quantities, fermentation pH and time on the breakdown of AFB_1_ were further studied. Microbes were generally temperature sensitive and changed at different temperatures. Therefore, the impact of fermentation temperature on AFB_1_ degradation was first studied. As can be seen in the results presented in Figure 4A, the isolated *E. faecium* HB2-2 demonstrated evident AFB_1_ degradation activity (from 47.0 to 53.5%) at all the examined fermentation temperatures in the range of 20–35 °C, which indicated *E. faecium* HB2-2 could grow and degrade AFB_1_ in a wide range of temperatures. *E. faecium* HB2-2 had the highest AFB_1_ degradation performance, reaching 53.5% at 32 °C, with the remaining AFB_1_ level dropping from 113.0 to 52.6 μg/kg following a 48 h fermentation period. Some previous investigations revealed that the AFB_1_-degrading activities of microorganisms were related to their fermentation temperature. *Bacillus licheniformis* CFR1 was observed to have a maximal AFB_1_ degradation activity at 37 °C [9], while *Bacillus subtilis* UTBSP1 isolated from pistachio nuts from Iran reduced AFB_1_ concentration to 80.5% after 48 h incubation at 30 °C [15].

The degradation rate of AFB_1_ was similarly impacted by the varied amounts of initial inoculum of *E. faecium* HB2-2. Therefore, the influence of the inoculation amount on the degradation of AFB_1_ was also studied. The experimental results are shown in Figure 4B; *E. faecium* HB2-2 observably displayed AFB_1_-degrading activity under all levels of initial inoculum, and the residual AFB_1_ was lowered considerably following treatment with *E. faecium* HB2-2. When the inoculation amount was 10% of NB medium, the AFB_1_ breakdown rate of HB2-2 strain was 58.9%, and the residual amount of AFB_1_ was reduced from 113.0 to 46.4 μg/kg. These results suggested that the optimal inoculation quantity of strain HB2-2 was 10% of NB medium.

The pH value of the medium not only impacts the growth of *E. faecium* HB2-2, but also impacts the AFB_1_-degrading activity. As demonstrated in Figure 4C, the AFB_1_ degradation of *E. faecium* HB2-2 was related with the pH values of fermentation medium. *E. faecium* HB2-2 demonstrated moderate AFB_1_ degradation ability (22.9–45.4%) at the pH levels of 2.0 to 7.0, whereas the degradation efficiency of *E. faecium* HB2-2 improved (49.8–69.3%) at pH values between 8.0 to 11.0, showing that the strain grew more successfully under alkaline conditions than in a neutral or acid environment. Hence, the ideal pH for strain HB2-2 was determined to be 10.0, the corresponding AFB_1_ degradation was 69.3%, and residual AFB_1_ concentration reduced from 113.0 to 34.7 μg/kg. These results were in keeping with the previous findings [44]. *Bacillus albus* strain YUN5 isolated from traditional Korean cuisine showed little AFB_1_ degradation at pH 2 and 4, but the greatest degradation of AFB_1_ at pH 10. These results suggested AFB_1_ degradation efficiency of the isolates altered substantially with fluctuation in pH values, and slightly alkaline circumstances may be advantageous to the breakdown of AFB_1_ by *E. faecium* HB2-2.

Fermentation length had a vital influence in the process. Thus, this inquiry explored the impact of fermentation length on AFB_1_ degradation of *E. faecium* HB2-2 in Figure 4D. The data revealed that when fermentation period prolonged from 24 to 120 h, the AFB_1_ degradation rate of *E. faecium* HB2-2 from 63.0% to 90.0%. At the ideal fermentation time of 96 h, the AFB_1_ breakdown rate by *E. faecium* HB2-2 reached 90.0%, with residual AFB_1_ dropping to 11.9 μg/kg. The result was consistent with the earlier research [44], where *Bacillus albus* YUN5 showed 37.9% AFB_1_ degradation on day 3, and 66.6% on day 7. In another investigation [50], *Bacillus subtilis* showed a 60% breakdown of AFB_1_ after 96 h of incubation. All of these investigations revealed the time-dependent degradation of AFB_1_, and showed that time duration was required to achieve maximum degradation.

### 3.4. Degradation of AFB_1_ by E. faecium HB2-2 in Peanut Meal

**Figure 5:**
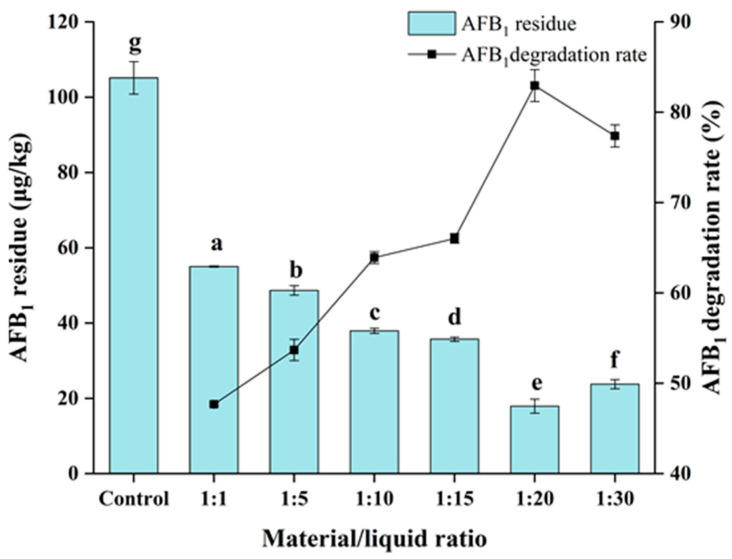
The degradation activity of AFB_1_ in peanut meal by *E. faecium* HB2-2. The peanut meal contaminated with AFB_1_ (105.1 μg/kg) was mixed with fermentation liquid at different material-to-liquid ratios (peanut meal/fermentation liquid, 1:1, 1:5, 1:10, 1:15, 1:20 and 1:30). Values in the bar graph represent the mean values ± SD of three independent experiments. Different lower-case letters in the same items indicate a significant result as determined by Duncan’s range test (*p* < 0.05).

To evaluate the AFB_1_ degradation ratio in the actual food and feed samples, *E. faecium* HB2-2 was further applied to moldy peanut meal containing AFB_1_. The AFB_1_ content of contaminated peanut meal used in this investigation was confirmed to be 105.1 μg/kg. The AFB_1_-contaminated peanut was employed as a fermentation substrate, combined with *E. faecium* HB2-2 at varying solid/liquid ratios (peanut meal/fermentation broth = 1:1 to 1:30), then it was incubated at 32 °C for 96 h. The residual AFB_1_ in peanut meal was extracted using immunoaffinity columns and quantified by an HPLC-fluorescent detector with pre-column derivatization. The results are shown in Figure 5; *E. faecium* HB2-2 revealed significant degrading activity (47.7–82.9%) of AFB_1_ in peanut meal, and the degradation rate showed a growing trend with the change in the solid/liquid ratio. When the ratio of material to liquid was 1:20, the breakdown rate of AFB_1_ in peanut meal was 82.9%, and the residual amount of AFB_1_ in peanut meal was reduced from 105.1 to 17.9 μg/kg. These results suggest the ratio of solid/liquid is an essential element impacting the microbial breakdown of AFB_1_, and adequate ratio of solid to liquid can boost the metabolism and proliferation of the strain. Similarly, an early study showed that the biological degradation technique of aflatoxins in peanut meal, which could approach 100% destruction of aflatoxins by combining heat treatment with anaerobic solid fermentation of *Streptococcus thermophilus* and *Lactobacillus delbrueckii subsp. Bulgaricus* [19]. In the investigation [15], *Bacillus subtilis* UTBSP1 isolated from pistachio nuts could eliminate 95% of AFB_1_ in the pulverized kernel of pistachio. All of these results revealed that microorganisms might be used for removing AFB_1_ from food and feed.

### 3.5. Different Cellular Fractions of E. faecium HB2-2 on AFB_1_ Degradation

**Figure 6:**
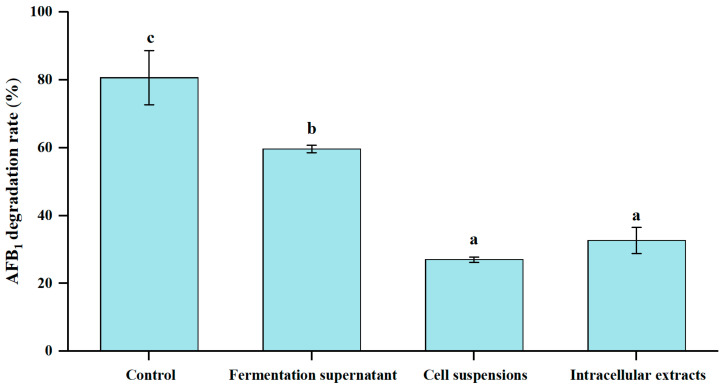
The AFB_1_ degradation activity of different cellular fractions of *E. faecium* HB2-2. The fermentation supernatant, cell suspensions and intracellular extracts were mixed with AFB_1_, respectively. *E. faecium* HB2-2 incubated with AFB_1_ served as a control. All groups were incubated at 32 °C for 96 h. Values in the bar graph represent the mean values ± SD of three independent experiments. Different small letters in the same items indicate a significant result as determined by Duncan’s range test (*p* < 0.05).

The effect of culture supernatants, cell suspensions and cell extracts of the isolated *E. faecium* HB2-2 on AFB_1_ degradation were further investigated. As shown in Figure 6, after incubation with AFB_1_ for 96 h, components of culture supernatants, cell suspensions and cell extracts showed varied breakdown activities for AFB_1_. The fermentation supernatant of *E. faecium* HB2-2 demonstrated considerably higher AFB_1_ degradation activity (59.7%) than cell suspension (27.0%) and cell extracts (32.7%). The results suggested that the breakdown of AFB_1_ by *E. faecium* HB2-2 largely depended on fermentation supernatant, and the AFB_1_ degradation components mainly existed in the extracellular supernatant produced in the fermentation process of *E. faecium* HB2-2. The result was congruent with other earlier publications. The culture supernatant of *Bacillus licheniformis* CFR1 showed a maximum AFB_1_ reduction of 93.6% after incubation, relative to 12.3% and 8.5% when treated with cell extract and viable cells [9], respectively. The bacteria cells of *Bacillus subtilis* UTBSP1 isolated from pistachio nuts revealed no ability to remove AFB_1_; however, the culture supernatant could significantly degrade 78.39% content of AFB_1_ [15]. Another study indicated that the culture supernatant of *Bacillus albus* YUN5 demonstrated 76.28% degradation for AFB_1_, whereas relatively minimal AFB_1_ degradation were found in the intracellular fraction and viable cells [44]. This research suggests that certain components found in the culture supernatant of *E. faecium* HB2-2 may play a role in the degradation of AFB_1_.

### 3.6. Effect of Heat Inactivation, Proteinase K and Proteinase K + SDS Treatments of Fermentation Supernatant on the AFB_1_ Degradation Activity 

**Figure 7:**
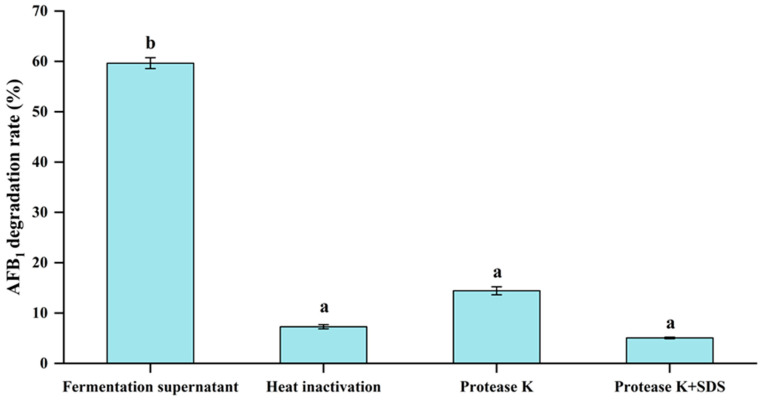
The AFB_1_ degradation activity of fermentation supernatant treated with heat inactivation, proteinase K and proteinase K+SDS. Values in the bar graph represent the mean values ± SD of three independent experiments. Different lower-case letters in the same items indicate a significant result as determined by Duncan’s range test (*p* < 0.05).

From the studies conducted above, it was obvious that the AFB_1_ degradation was greatest in the culture supernatant in comparison with cell and cell extracts of *E. faecium* HB2-2. Hence, the fermentation supernatant of strain HB2-2 was supplied with different treatments to detect the components that were responsible for AFB_1_ degradation. The AFB_1_ breakdown activity of fermentation supernatant treated with heat, proteinase K, and SDS were examined, respectively, and the findings were displayed in Figure 7. Surprisingly, the AFB_1_ degradation rate of fermentation supernatant treated with heat inactivation was only 7.3%, which was dropped considerably compared with the untreated supernatant with 59.7% AFB_1_ degradation activity. In addition, the breakdown rates of the fermentation supernatant treated with protease K and protease K+SDS were 14.4% and 5.1%, respectively. This result clearly suggested degradation of AFB_1_ was probably related to the enzyme generated by *E. faecium* HB2-2 in the culture supernatant. After protease K, protease K+SDS and heat treatment, the protease secreted by strain HB2-2 was denatured, and AFB_1_ degradation activity of the fermentation supernatant was lost. These findings are in keeping with a previously published report, revealing that the AFB_1_ degradation in the culture supernatant of *Bacillus licheniformis* CFR1 dropped considerably from 88.6% to 26.9% following treatment with proteinase K [44]. Another study found that 88.0% of AFB_1_ were degraded by the culture supernatant of *Trichoderma reesei*; however, AFB_1_ degradation fell dramatically to 69.6% following treatment with proteinase K, and declined to 29.4% after treatment with proteinase K plus SDS [51]. Similar results were also reported in other AFB_1_-degrading bacteria, such as *Mycobacterium fluoranthenivorans*, *Bacillus velezensis* DY3108, *Staphylococcus* sp. [18,52,53]. These data revealed that enzymes or proteins played a crucial role in AFB_1_ degradation. However, more studies are required to identify the enzyme or enzyme systems responsible for the biodegradation.

### 3.7. Identification of the AFB_1_ Degradation Products 

**Figure 8:**
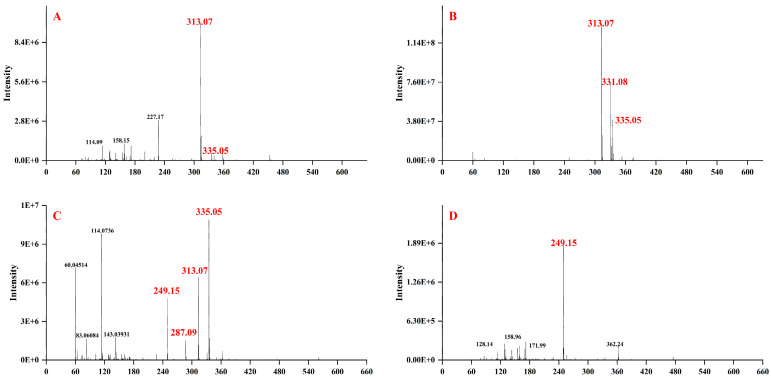
Analysis and identification of AFB_1_ and its degraded products. (**A**) The mass spectra of AFB_1_ in the control group untreated with *E. faecium* HB2-2; (**B**–**D**) the mass spectra of AFB_1_-degraded products by *E. faecium* HB2-2.

**Figure 9:**
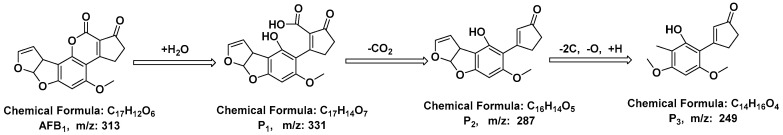
The hypothetical degradation pathway of AFB_1_ based on LC–MS analysis of the degraded products.

LC–MS analysis is often used to examine the degradation products of AFB_1_. As indicated in Figure 8A, the ion peaks at m/z 313 [M+H]^+^ and 335 [M+Na]^+^ related to AFB_1_ were discovered in the mass spectrum of the control group (NB media with AFB_1_), these distinctive peaks were also founded in the mass spectrum of AFB_1_ treated with *E. faecium* HB2-2 (Figure 8B), and are attributable to residual AFB_1_. These characteristic peaks results were in agreement with shu et al. who discovered particular ion peaks of AFB_1_ with m/z 313, 335, and 647 in the complete mass spectrum of AFB_1_ degradation products degraded by *Bacillus velezensis* DY3108 [52]. Wang et al. observed the specific ion peaks of AFB_1_ at m/z 313 [M+H]^+^ and m/z 335 [M+Na]^+^ in the fermentative mixture by fast biodegradation of AFB_1_ by metabolites of *Fusarium* sp. WCQ3361 [27]. Additionally, LC–MS analysis indicated multiple ion peaks at distinct m/z values related to complicated degradation products of AFB_1_ in the mass spectrum of the *E. faecium* HB2-2-treated group. The primary ion peaks of products at m/z 331 (P_1_, C_17_H_15_O_7_), 287 (P_2_, C_16_H_15_O_5_), 249 (P_3_, C_14_H_17_O_4_) in Figure 8B–D). Subsequently, the likely degraded products of AFB_1_ were postulated and a tentative degradation pathway of AFB_1_ was created based on the tiny fragments. As illustrated in Figure 9, the hydrolysis reaction of the lactone ring in AFB_1_ structure catalyzed by enzymes generated by HB2-2 resulted in a product P_1_ with m/z 331 (C_17_H_14_O_7_). The hydrolysis was then followed by decarboxylation of the open lactone ring, and P_1_ was further transformed into compound P_2_ at m/z 287 (C_16_H_14_O_5_) by cleaving the lactone ring by the removal of CO_2_. Subsequently, P_2_ product was further converted into compound P_3_ with m/z 249 (C_14_H_16_O_4_) by deleting the furan rings of AFB_1_. These LC–MS data demonstrated the susceptibility of the AFB_1_ lactone ring and furan rings against *E. faecium* HB2-2. Some prior research claimed the lactone ring structure in AFB_1_ is easily hydrolyzed, Eshelli et al. disclosed the breakdown of the terminal lactone ring of AFB_1_ to create a carboxylic acid product with a m/z value of 331, and the product with a m/z value of 287 after decarboxylic reaction [54]. Wang et al. reported an AFB_1_-degrading bacteria *Escherichia coli* CG1061 isolated from chicken cecum, the products of AFB_1_ were examined by UPLC Q-TOF MS and the m/z ion peaks at 287 was identified as the probable degradation product [55]. A similar process, in accordance with our results, was observed in other studies showing the biological degradation of AFB_1_ by microorganism [19,56,57,58].

### 3.8. Cytotoxicity Assay of the AFB_1_ Degradation Products

**Figure 10:**
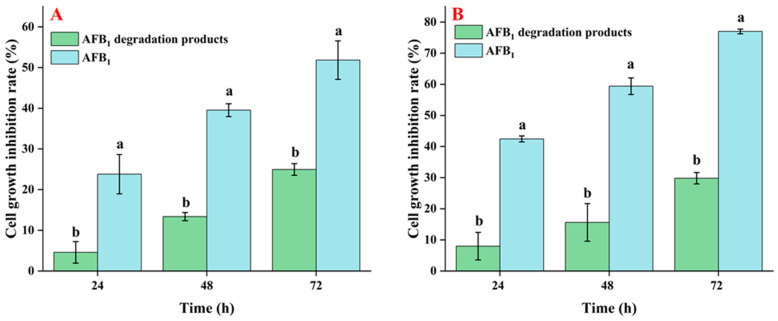
Cytotoxicity assay of the degraded products. The toxicity of the AFB_1_ and its degraded products were determined by examining the cell inhibition rates of (**A**) HepG2 and (**B**) RAW264.7 cells at different times. Values in the bar graph represent the mean values ± SD of three independent experiments. Different lower-case letters in the same items indicate a significant result as determined by Duncan’s range test (*p* < 0.05).

**Figure 11:**
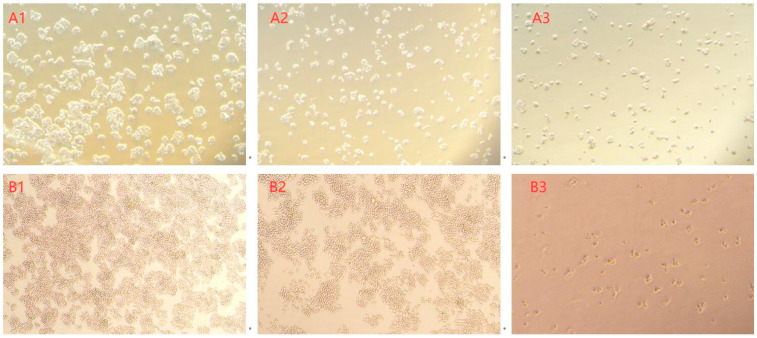
The effects of AFB_1_-degraded products on cell morphology. (**A1**) The morphology of HepG2 cells in control group (without AFB_1_ and its degraded products); (**A2**) the morphology of HepG2 cells treated with degraded products; (**A3**) the morphology of HepG2 cells treated with AFB_1_; (**B1**) the morphology of RAW264.7 cells in control group (without AFB_1_ and its degraded products); (**B2**) the morphology of RAW264.7 cells treated with degraded products; (**B3**) the morphology of RAW264.7 cells treated with AFB_1_.

In this investigation, the toxicity of AFB_1_ and its degradation products reduced by *E. faecium* HB2-2 was tested by cell viability of HepG2 and RAW264.7 cells. AFB_1_ residues present in NB media untreated with strain HB2-2, as well as the degraded products of AFB_1_ in fermentation supernatant treated with *E. faecium* HB2-2, were isolated utilizing immunoaffinity columns. Subsequently, they were cultured with HepG2 and RAW264.7 cells for 24, 48, and 72 h. As shown in Figure 10, AFB_1_ isolated from NB demonstrated cytotoxicity rates of 25.8%, 39.5%, and 45.8% against HepG2 cells after 24, 48, and 72 h of treatment, respectively. Degradation products of AFB_1_ treated with *E. faecium* HB2-2 demonstrated only 6.2%, 13.4%, and 23.5% cytotoxicity on HepG2 cells at 24, 48, and 72 h, respectively. The same trend was found with RAW264.7 cells, untreated AFB_1_ displayed cytotoxicity rates of 42.5%, 59.4% and 76.9% against RAW264.7 cells at 24, 48, and 72 h, respectively. In contrast, the cell inhibition rates of degraded products of AFB_1_ treated with *E. faecium* HB2-2 were only 8.0%, 15.6% and 29.8% at 24, 48, and 72 h, respectively. The cellular morphology was further observed, and the cell shape in the AFB_1_-treated group was changed significantly compared with the degraded products group (Figure 11). These results indicated higher cellular toxicity of AFB_1_ extracted from untreated group compared to the degradation products of AFB_1_ treated with *E. faecium* HB2-2. Which suggested that the AFB_1_ was degraded by HB2-2, and the toxicity of products was lowered compared to the parent AFB_1_. These findings were associated with the LC-MS results revealing the efficiency of HB2-2 towards the toxicogenic difuran and lactone rings in AFB_1_. These findings were corroborated by Kumar et al., who demonstrated that the cytotoxicity of AFB_1_-degraded products by *Bacillus albus* YUN5 was significantly reduced compared to the untreated AFB_1_ sample [44]. Some additional microorganisms have shown the identical effect to degrade AFB_1_ to some small molecules with minimal cytotoxicity, such as *Bacillus velezensis* [52], *Streptococcus thermophilus* [19], and *Fusarium* sp. WCQ3361 [27].

## 4. Conclusions

In this work, a novel bacterium HB2-2 was isolated for degradation of AFB_1_, and it was recognized as *Enterococcus faecium* by combining morphology, biochemical features and 16S rRNA sequence analysis. The isolate showed remarkable AFB_1_ degradation ability under different conditions, including varying temperatures, bacterial quantities, fermentation times, and pH values, indicating its potential application in diverse environmental conditions. Notably, *E. faecium* HB2-2 degraded 82.9% of AFB_1_ in peanut meal, reducing the residual AFB_1_ concentration from 105.1 to 17.9 μg/kg. The AFB_1_ degradation ability of *E. faecium* HB2-2 was found to rely on the fermentation supernatant, and the degradation factor may be a biological enzyme produced during the fermentation process. The products of AFB_1_ degradation by *E. faecium* HB2-2 were analyzed by LC-MS, possible degradation products were postulated, and a tentative AFB_1_ degradation pathway was established based on the identified degradation products. The proposed degradation mechanism suggests that *E. faecium* HB2-2 targets the difuran ring and lactone ring of the AFB_1_ molecule. Additionally, cytotoxicity experiments on HepG2 and RAW264.7 cells indicated that the toxicity of the degradation products was significantly lower than that of the parent AFB_1_. *E. faecium*, a naturally occurring probiotic component widely adopted in foods and animal feeds, proves beneficial in enhancing animal health. These suggest the reliability and safety of using *E. faecium* HB2-2 as an effective method for the degradation of AFB_1_ in food and feed processing.

## Figures and Tables

**Table 1:** The physiological and biochemical characteristics of strain HB2-2.

Experimental Items	Results	Experimental Items	Results
Gram stain	+ ^a^	Oxidase	-
Methyl red test	-	Starch hydrolysis	+
Anaerobic growth	-	Tween 20	+
Citrate utilization	-	Sucrose utilization	+
Propionate utilization	+	Glucose utilization	+
Lactose utilization	+	2% NaCl	+
7% NaCl	+	Growth at 4 °C	-
Growth at 28 °C	+	37 °C growth	-
V-P test	-	pH6	+
Contact enzymes	+	pH10	+

^a^ Symbols: +, positive; -, negative.

## Data Availability

The original contributions presented in the study are included in the article/supplementary material, further inquiries can be directed to the corresponding author.

## Supplementary Materials

The following supporting information can be downloaded at https://www.mdpi.com/article/10.3390/foods13121887/s1. Figure S1. The quantity of AFB1 was measured using an HPLC system; Figure S2. The Standard curve of AFB1 measured by HPLC.
