# Supplementary material for: Biological Detoxification of Aflatoxin B1 by Enterococcus faecium HB2-2
Source: Foods. 2024 Jun 15;13(12):1887. doi: 10.3390/foods13121887 (PMC11202875; doi:10.3390/foods13121887)
Supplement: Supplementary file 1 [file foods-13-01887-s001.zip › foods-3029919-supplementary.pdf]

Supporting information

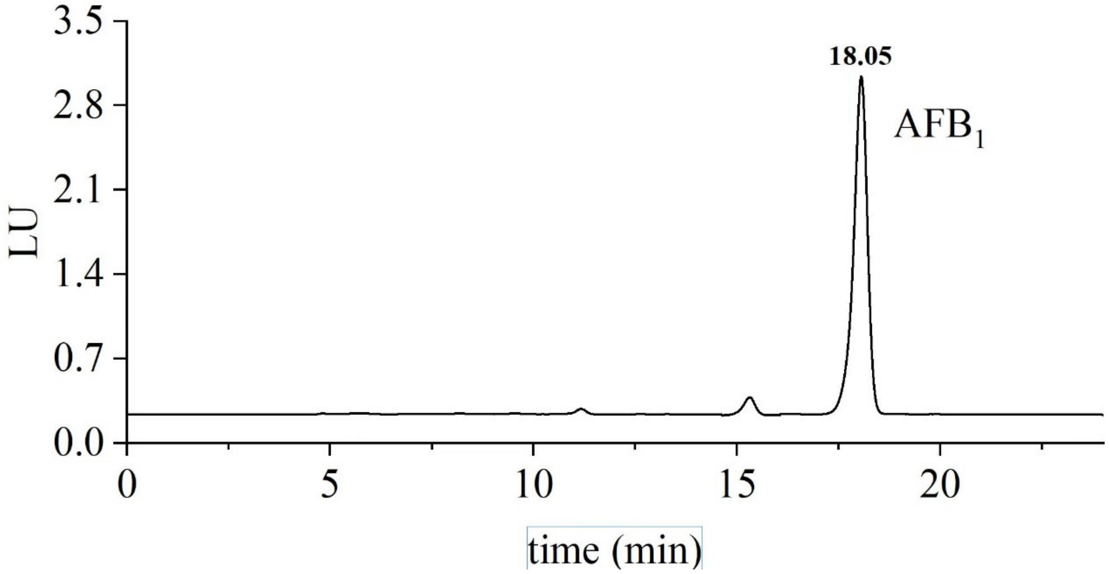

Figure S1. The quantity of AFB_1 was measured using an HPLC system

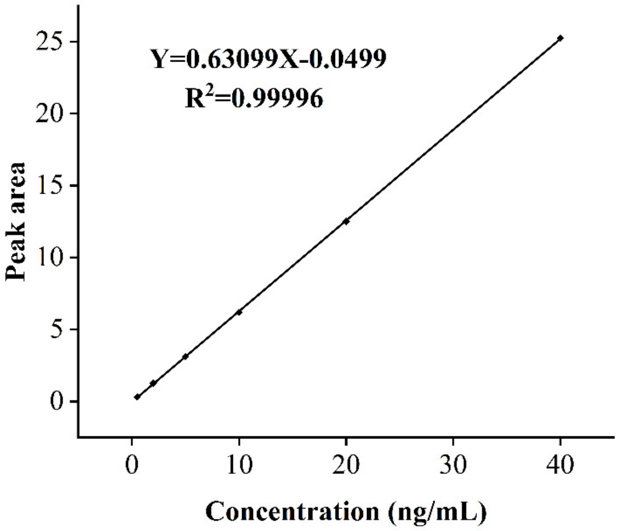

Figure S2. The Standard curve of AFB_1 measured by HPLC
